# Effect of Lignosulphonate-Based Biostimulants and Grafting on Watermelon Plant Performance Under Open-Field Conditions for Two Consecutive Years

**DOI:** 10.3390/plants15182887

**Published:** 2026-09-21

**Authors:** Pietro Bellitto, Fabiana Mancuso, Leo Sabatino, Georgia Ntatsi, Salvatore La Bella, Beppe Benedetto Consentino

**Affiliations:** 1 Department of Agriculture, Food and Forestry Sciences, University of Palermo, 90128 Palermo, Italy; pietro.bellitto@unipa.it (P.B.); fabiana.mancuso@unipa.it (F.M.); salvatore.labella@unipa.it (S.L.B.); beppebenedetto.consentino@unipa.it (B.B.C.); 2 Laboratory of Vegetable Production, Department of Crop Science, Agricultural University of Athens, 11855 Athens, Greece; ntatsi@aua.gr

**Keywords:** herbaceous grafting, *Citrullus lanatus*, productive traits, fruit quality, lignosulphonate, physiological traits

## Abstract

Lignosulfonate-based biostimulants (LS) are recognized to play a role as chelating and careering agents for micronutrients. Concomitantly, vegetable grafting is a recognized technique with which to improve yield and fruit quality and increase plant tolerance to abiotic and biotic distresses. The objective of this study was to evaluate how the integration of these complementary approaches could affect agronomic attributes, colorimetric parameters, qualitative and nutritional characteristics, and the physiological traits of watermelon plants. The combined effects of LS biostimulant and grafting (non-grafting, self-grafting, or grafting onto ‘RS 841’ rootstock) were evaluated in a two-year (2024 and 2025) open-field study. Plots treated with LS biostimulant yielded 5% more than control plots, whereas grafted plots yielded between 12 and 13% more than self-grafted and non-grafted plots, respectively. In addition, biostimulant treatment reduced physiological traits such as stomatal conductance and transpiration, whereas grafting only enhanced stomatal conductance. Furthermore, LS biostimulant increased β-carotene (+25%), lycopene (+21%) and carotenoid (+21%) contents, while grafting had the same effect and increased β-carotene (+55%), lycopene (+50%) and carotenoids (+48%) when compared with the control treatment. Results indicated that the effects of the biostimulant and grafting technique were dependent on the year. Accordingly, this study suggested that for yield, physiological traits, quality, nutritional characteristics and colorimetric parameters, the year of experimentation should be no taken into consideration, whereas for percentage of fruit dry matter, solid soluble content, pulp firmness, and epicarp thickness, the year of experimentation should be taken into consideration in order to improve crop quality.

## 1. Introduction

Watermelon (*Citrullus lanatus* (Thunb.) Matsum. & Nakai) is among the most widely produced fruiting vegetables worldwide, with global production exceeding 100 million tons annually [1]. It is a source of water and bioactive compounds that are beneficial to human health, such as carotenoids (lycopene and β-carotene), volatile compounds (VOCs), organic acids (citric and glutamic acid), and amino acids (citrulline and arginine) [2,3,4,5,6]. Moreover, it contains high quantities of polyphenolic compounds that have anticancer and antioxidant properties [7]. Thus, the consumption of watermelon fruits can also decrease the occurrence of chronic human diseases via the reduction of reactive oxygen species buildup [8].

Due to the expansion of cultivated areas and the growing demand for watermelon fruits, production is facing challenges linked to biotic and abiotic stresses. These challenges bring to light agronomic and plant health issues, which negatively affect the yield and quality of this crop in terms of productivity and sustainability. Among the main limitations, soil degradation and the presence of pathogens are the most important. In this regard, *Fusarium oxysporum* f. sp. *niveum*, the pathogen responsible for tracheofusariosis and other soil-borne pathogens such as *Verticillium dahliae*, *Phytophthora* spp., and *Meloidogyne* spp. (root-knot nematodes), can reduce productivity by up to 80% under high infection pressure [9]. Moreover, cultivation practices adopted for watermelon contribute to soil degradation, a phenomenon that is particularly evident in intensive cropping systems [9,10]. In addition, watermelon production is constrained by a wide range of factors, such as mites [11], water availability [12], salinity [13,14], and temperatures, particularly low temperatures [15,16]; all of these factors can potentially compromise yield and quality. Furthermore, prolonged monoculture and the intensification of agricultural practices have exacerbated the situation, making it necessary to adopt innovative management approaches [5].

Herbaceous grafting has established itself as a sustainable and highly effective agronomic technique for controlling soil-borne pathogens, improving tolerance to abiotic stress, and enhancing watermelon yield [17]. Grafting is a propagation technique that involves joining two plant individuals—the rootstock and the scion—to combine the advantageous characteristics of both. Studies have shown that the intrinsic and extrinsic quality traits of vegetable fruits can be influenced in various ways by the genotypes of the grafted plants (both the scion and the rootstock), by the respective rootstock–scion combinations and their compatibility, and by the growing conditions [17,18]. In watermelon production, an interspecific hybrid of *Cucurbita maxima × Cucurbita moschata* (F_1_ RS 841) is often chosen as rootstock due to its high affinity with other cucurbits, vigorous root system, and tolerance to many soil-borne pathogens (e.g., *Fusarium oxysporum* f.sp *niveum* and *Verticillium dahliae*) [19] (Hultron et al., 2007).

The use of plant biostimulants (microbial and non-microbial) is another promising innovative approach through which to enhance plant performance under different growing conditions [20,21,22]. Lignosulphonates (LSs) are non-microbial biostimulants obtained as by-products of the pulp and paper industry, resulting from the processing of lignocellulosic biomass using the sulphite process [23]. It is well known that this environmentally friendly and cost-effective material can improve soil quality and the efficacy of fertilisers, whilst replacing or reducing the use of potentially (eco)toxic organic or inorganic substances [24]. Indeed, LSs act as biostimulants by modulating the behaviour of fertilisers, offering benefits in fertiliser-related applications. It has been demonstrated that LSs can enhance plant growth and development [25]. However, there is often a lack of long-term applications and studies, and further research is needed to develop products that are economically competitive with commercial bulk products.

To the best of our knowledge, there are no reports on the combined application of grafting and LS on watermelon plants cultivated in the open field. Our hypothesis is that herbaceous grafting may modulate the efficacy of LSs, further improving the yield, quality and physiological parameters of watermelon plants grown in open fields. In this regard, grafting onto vigorous rootstocks can modify the root system growth, while LS can improve nutrient availability. The synergy of grafting and LS could further ameliorate plant growth, mineral nutrition and physiological performance. Despite the potential use of these two agronomic approaches, the effects of their combined application remain understudied. Therefore, the aim of this study was to evaluate the interactive effect between a commercial lignosulphonate-based biostimulant (STATIA^®^) and different grafting combinations (non-grafted, self-grafted or grafted onto the F_1_ RS 841 rootstock) on watermelon plants grown in the open field for two years. Analysis of the data obtained, which is compared with existing data in the literature, could lead to the development of a cultivation protocol suitable for Mediterranean farms and to the foundations for further studies aiming to apply this protocol on a large-scale basis.

## 2. Results

### 2.1. Climatic Data

**Figure 1:**
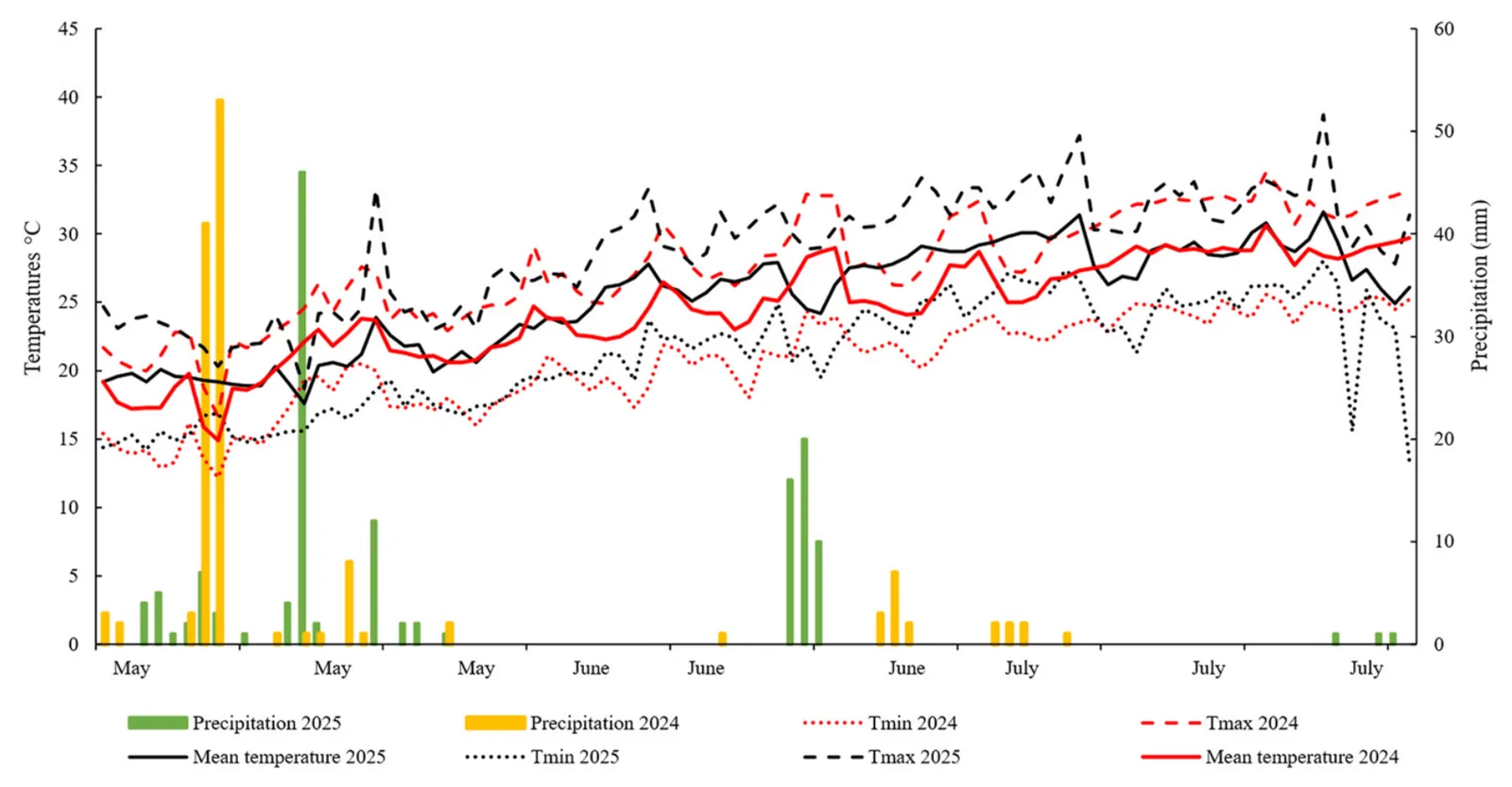
Dynamics of mean, minimum, and maximum temperatures during 2024 (red continuous and discontinuous lines) and 2025 (black continuous and discontinuous lines) and mean precipitation during the 2024 (yellow bars) and 2025 (green bars) growing seasons.

The hydrothermal conditions for all growing seasons are shown in Figure 1. The dynamics of mean temperatures between May and July were similar between growing seasons. During the reproductive stage of the crop (June–middle July), mean temperatures were higher in 2025 than in 2024, at 27 °C and 26 °C, respectively. The black arrows indicate short-term temperature events during May and July. The dynamic of precipitation was also similar between growing seasons, although total precipitation was slightly higher in 2025 (141 mm) than in 2024 (136 mm) over the May–July period. All these data indicate that the two growing years were characterized by similar climatic conditions, although temperatures were slightly higher in 2025. The recorded conditions during a critical phase, such as the reproductive stage, could affect physiological processes, potentially influencing the plant response between the two cultivation cycles. Overall, climatic data provided an environmental background to assess the potential beneficial effects of biostimulants and grafting across different growing years.

### 2.2. ANOVA Output

A three-way ANOVA was performed for the LS biostimulant, grafting technique, year, and their interactions on marketable yield and the fruits’ components, color parameters, firmness, solid soluble content, dry matter, stomatal conductance, transpiration and carotenoids (including β-carotene and lycopene). The ANOVA revealed highly significant B × G interaction effects on pulp firmness, peel firmness, soluble solids content (SSC), fruit dry matter, stomatal conductance, transpiration and β-carotene; B × Y interaction significantly affected marketable yield, L* pulp, pulp firmness, peel firmness, SSC and fruit dry matter; and G × Y interaction meaningfully modulated L* pulp, a* pulp, peel firmness, epicarp thickness, SSC and stomatal conductance. In addition, ANOVA revealed significant B × G × Y interaction for pulp firmness, peel firmness, epicarp thickness, SSC and dry matter (Table S1; see Supplementary section). These results indicate that the effectiveness of LS applications on plant fruit quality and physiological traits depended on grafting strategy and on cultivation year.

### 2.3. Productive and Physical Quality Features: Marketable Yield, Fruit Firmness, Epicarp Thickness, and Dry Matter of Watermelon

Table 1 shows the main effect of LS biostimulants and grafting on the number of marketable fruit and average weight of marketable fruits.

Biostimulant did not significantly affect number of marketable fruits and average weight of marketable fruits. However, irrespective of biostimulant and year, grafted plants showed the highest number of marketable fruits and average weight of marketable fruits (Table 1). The growing year had no significant effect on number of marketable fruits, whereas it significantly influenced the average marketable fruit weight, with the highest values recorded in plants from the second cultivation year.

**Figure 2:**
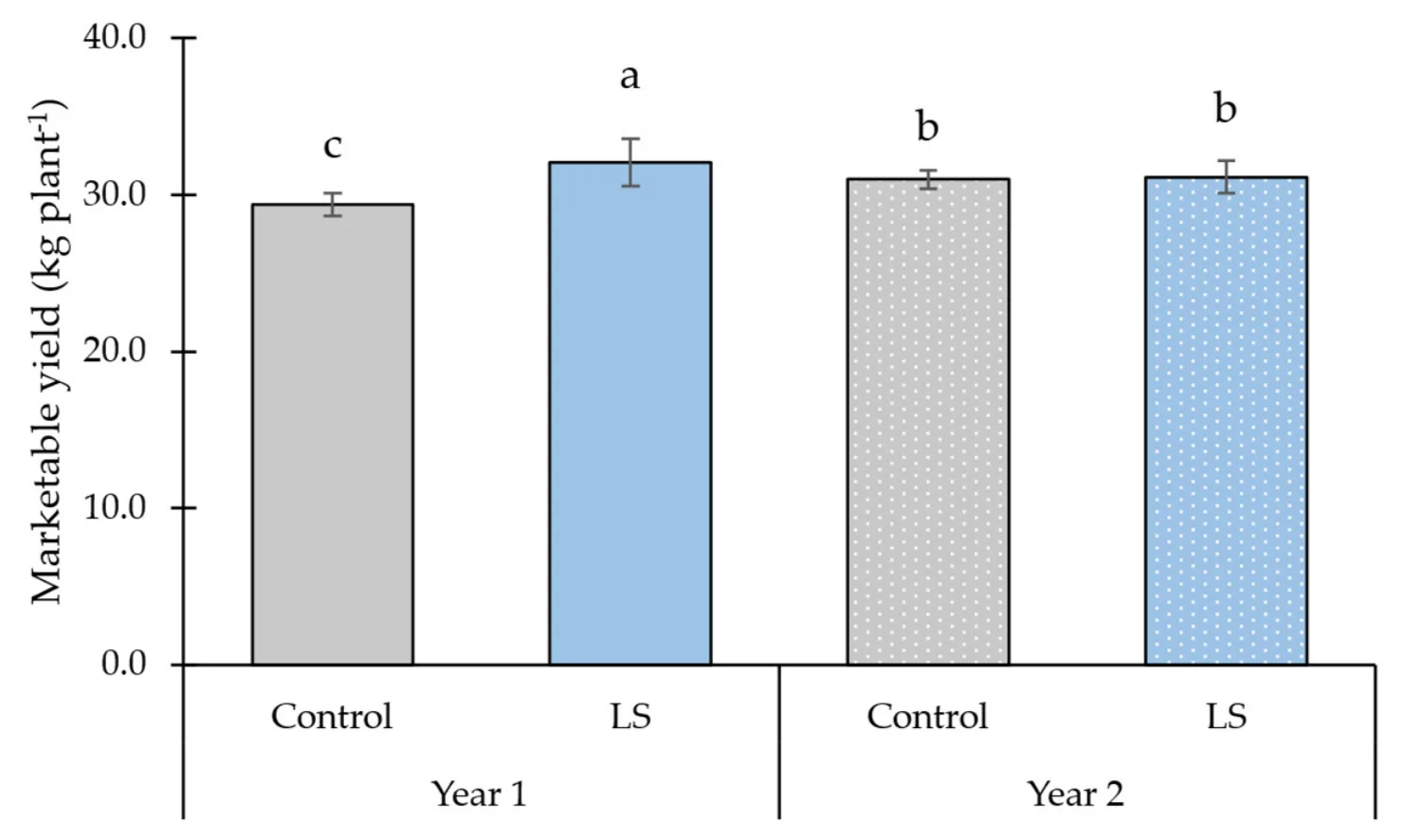
Effect of biostimulant and year on watermelon marketable yield. Different letters indicate different means according to Tukey’s HSD test at *p* ≤ 0.05. LS: lignosulphonate-based biostimulant; Y1; 2024; Y2: 2025. Bars indicate means ± SE.

Regarding the B × Y interaction, the highest marketable yield was recorded in plots treated with LS biostimulant during year 1. No further differences were observed for control and LS biostimulant treatments during year 2, nor in control plots of year 1 (Figure 2).

**Figure 3:**
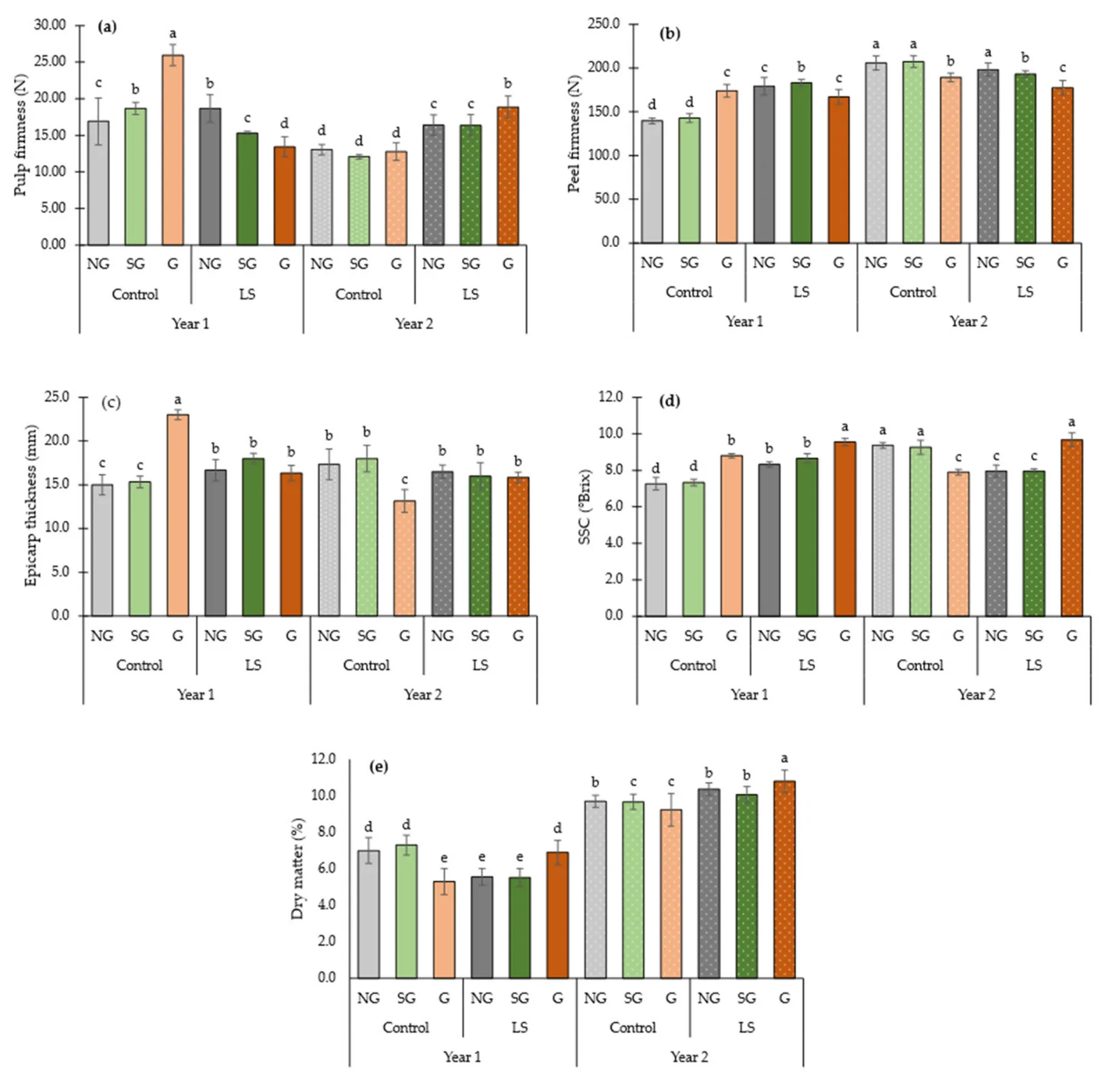
Effect of biostimulant grafting and year on watermelon pulp firmness (**a**), peel firmness (**b**), epicarp thickness (**c**), soluble solids content (SSC) (**d**), and dry matter (**e**). Different letters indicate different means according to Tukey’s HSD test at *p* ≤ 0.05. LS: lignosulphonate-based biostimulant; NG: non-grafted; SG: self-grafted; G: grafted; Y1; 2024; Y2: 2025. Bars indicate means ± SE.

Figure 3 shows the significant triple interaction among lignosulphonate-based biostimulant, grafting, and year for pulp and peel firmness, epicarp thickness, solid soluble content, and fruit dry mass.

In year 1, grafting increased pulp firmness in control plots (>25 N), whereas the lowest pulp firmness values were observed in grafted, self-grafted and non-grafted plots during year 2 (~12.5 N) and in LS × grafted plots in year 1 (Figure 3a). In addition, Figure 3b shows that the highest values for peel firmness were observed in non-grafted and self-grafted control plots and in non-grafted LS plots (~200 N) in year 2, whereas the lowest values were observed in non-grafted and self-grafted control plots of the year 1 (<150 N). Moreover, Figure 3c shows that the highest epicarp thickness values were found in grafted control plots of year 1 (24 mm), whereas the lowest values were found in grafted control plants of year 2 (14 mm). For soluble solid content (Figure 3d), results showed that the highest values were recorded in LS-grafted plots of year 1 and in treatment combinations of year 2 (NG × control, SG × control and G × LS). The lowest values were found in control-grafted and self-grafted plots of the first year (<8°Brix). Regarding fruit dry matter percentage, plants cultivated during year 2 that were grafted and treated with LS revealed the highest values, whereas the lowest ones were recorded in year 1 G × control, NG × LS, and G × LS combinations (Figure 3e).

### 2.4. Colorimetric Traits: Brightness (L*), Redness (a*), and Yellowness (b*)

**Figure 4:**
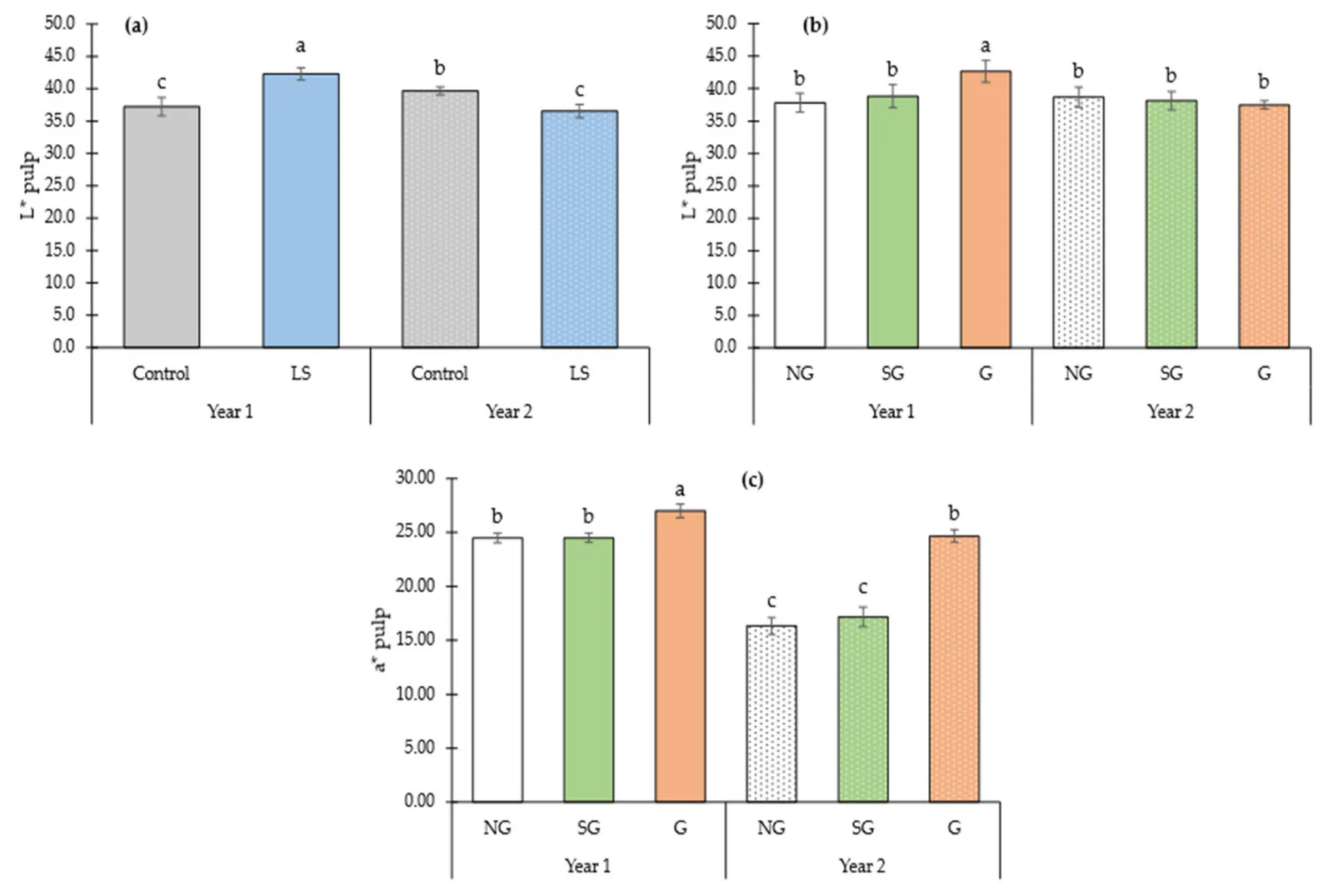
Effect of biostimulant grafting and year on watermelon L* (**a**,**b**) and a* (**c**) colorimetric parameters. Different letters indicate different means according to Tukey’s HSD test at *p* ≤ 0.05. LS: lignosulphonate-based biostimulant; NG: non-grafted; SG: self-grafted; G: grafted; Y1; 2024; Y2: 2025. Bars indicate means ± SE.

When the B × Y interaction (Figure 4a) was considered, the highest pulp brightness values were registered in LS-biostimulated plots during year 1 (42.0), whereas the lowest L* values were found in the LS-treated plots of year 2.

Moreover, taking into consideration the G × Y interaction, the highest values were observed in grafted plots of year 1, differing from the rest of the treatments which had the lowest values (Figure 4b). Regarding redness, grafting treatments during year 1 gave the highest values (~27.5), in contrast with non-grafted and self-grafted treatments in year 2, which gave the lowest results (~15).

The ANOVA revealed that biostimulant treatments did not modify b* color parameter. Among grafting treatments, grafted plots showed significantly higher b* values than self-grafted and non-grafted plots. Concerning year, b* showed higher values in year 1 than in year 2 (Table 2).

### 2.5. Qualitative and Nutritional Parameters: β-Carotene, Lycopene, and Carotenoid Content

**Figure 5:**
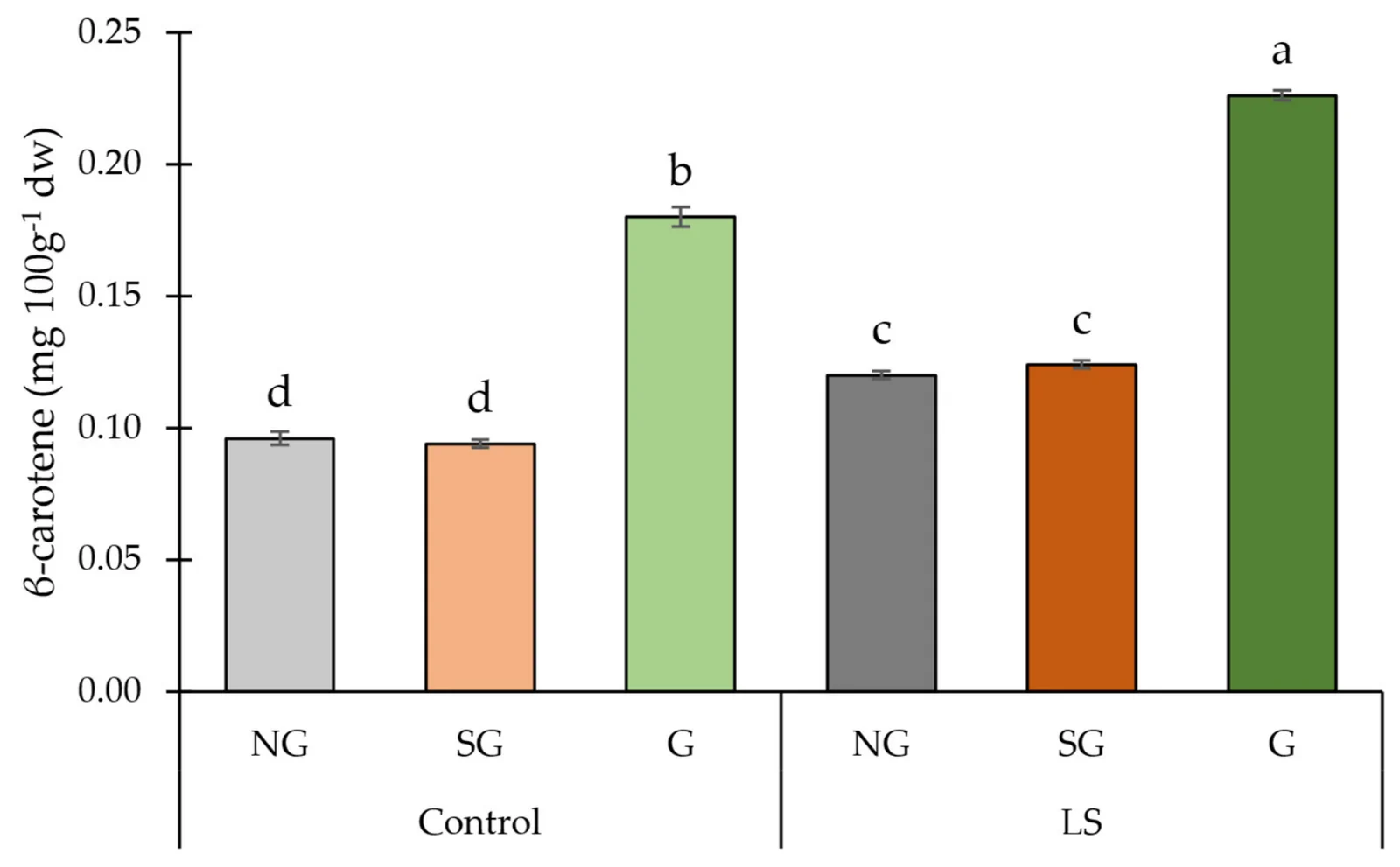
Effect of biostimulant and grafting on watermelon β-carotene concentrations. Different letters indicate different means according to Tukey’s HSD test at *p* ≤ 0.05. LS: lignosulphonate-based biostimulant; NG: non-grafted; SG: self-grafted; G: grafted. Bars indicate means ± SE.

When the B x G interaction was significant, the highest β-carotene values were observed in grafted and biostimulated plots (>0.20); in opposition, non-grafted and self-grafted control plots displayed the lowest β-carotene values (<0.10) (Figure 5).

The main effects of biostimulant, grafting, and year on lycopene and carotenoids are reported in Table 3.

Data showed that biostimulant treatments increased lycopene by 21% and carotenoid content by 21% (expressed as mg 100 g^−1^ of dry weight) when compared with control treatments. In addition, grafting treatments increased lycopene by 50% and carotenoid content by 48% when compared with control treatments (non-grafted). Regarding year, no differences were found between year 1 and 2 for lycopene and carotenoid content (Table 3).

### 2.6. Physiological Traits: Stomatal Conductance and Transpiration

**Figure 6:**
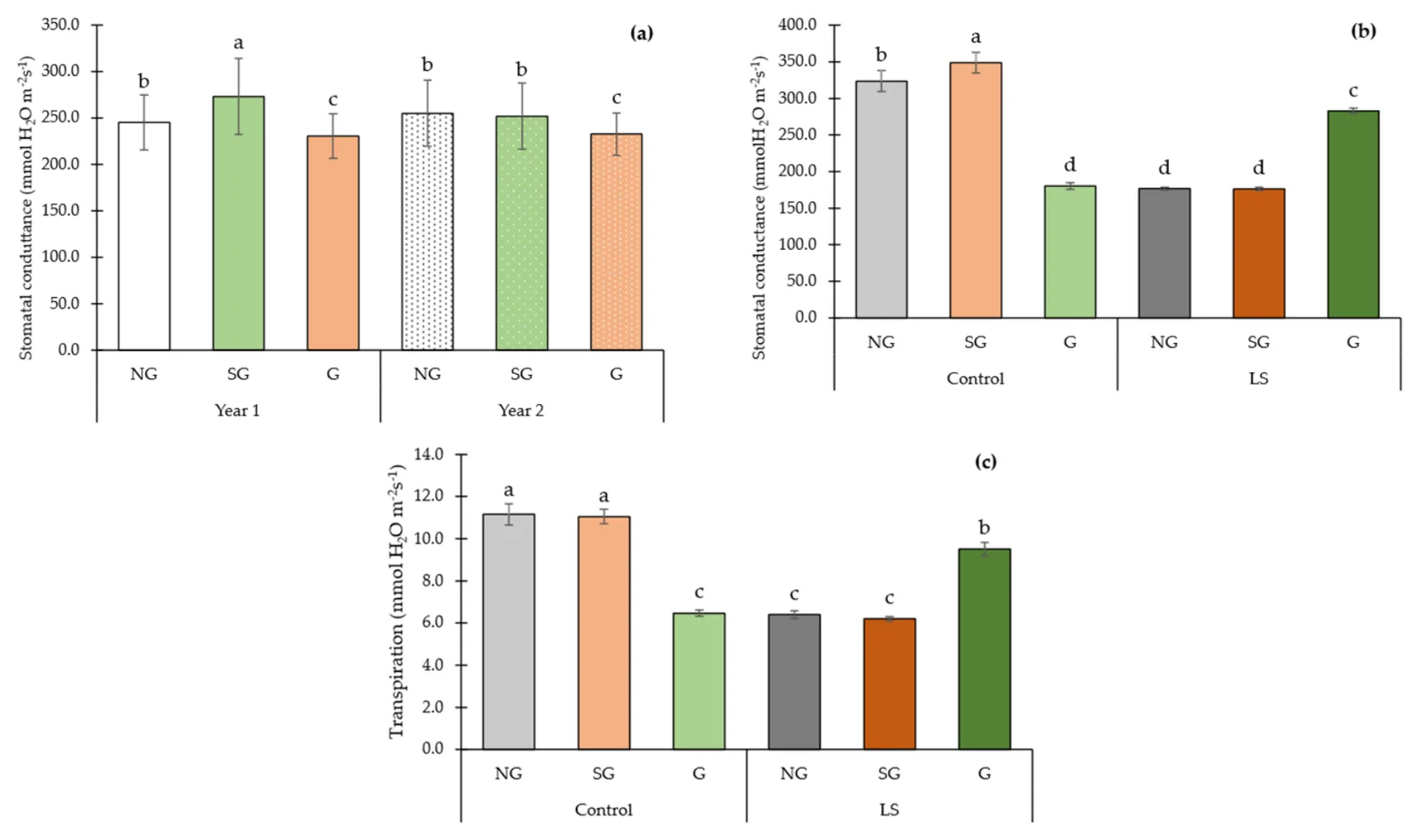
Effect of biostimulant, grafting, and year on watermelon plant stomatal conductance (**a**,**b**) and transpiration (**c**). Different letters indicate different means according to Tukey’s HSD test at *p* ≤ 0.05. LS: lignosulphonate-based biostimulant; NG: non-grafted; SG: self-grafted; G: grafted. Bars indicate means ± SE.

Figure 6a displays the G × Y interaction effect on stomatal conductance, whereas the G × B combinations for stomatal conductance and transpiration are reported in Figure 6b,c.

The highest values for stomatal conductance were achieved during year 1 in self-grafted plots (~350), whereas the lowest ones were observed in grafted plots of year 1 and in those of year 2 (Figure 6a). For the G × B interaction, conductance peaked in self-grafted control plots, whereas the lowest values were found in grafted control plants and in non-grafted and self-grafted plots treated with LS (Figure 6b). Higher transpiration values were recorded in non-grafted and self-grafted control plants, followed by those grafted and treated with LS; all the other treatment combinations gave the lowest values (Figure 6c).

## 3. Discussion

The current research assessed the use of grafting and lignosulfonate on watermelon grown in open-field conditions for two consecutive cultivation cycles, investigating productive, qualitative, and physiological traits.

### 3.1. Productive Traits

The differences observed between the two cultivation years suggested that the overall plant productivity and marketable yield were strongly plant season-dependent, in turn influencing plant responses to biostimulants. Moreover, LS treatment increased marketable yield compared to control plots in both cultivation cycles. These results are in line with those of Ertani et al. [23] and Docquier et al. [24], who evidenced an increase in growth and productive traits of LS-treated plants. As reported by Ertani et al. [25], the higher yield of plants supplied with lignosulfonates could be directly related to their ability to promote root development, influencing the hormonal balance and modulating some growth regulators, such as indoleacetic acid (IAA) and nitric oxide (NO). Furthermore, the application of LS can enhance the activity of the H^+^-ATPase root membrane and modulate the electrochemical gradient, resulting in a higher nutrient use efficiency [26]. Additionally, according to the results of the present study, grafted plants showed the highest marketable yield compared to self-grafted or non-grafted plants. The results agree with those of Kyriacou et al. [18] and Davis et al. [27], who studied the effects of grafting on *Cucurbitaceae*. This effect could be attributed primarily to the functionality of the root system, which is more developed in grafted plants than in non-grafted ones, ensuring better absorption of water and nutrients and, consequently, higher rates of photosynthesis and yield compared to non-grafted plants [28].

Interestingly, data indicated that grafting significantly enhanced the number of marketable fruits. Although strongly related to the genotype, the fruit number could be influenced by the scion-rootstock affinity and by the vigor of the rootstock. As reported by Alan et al. [29], the number of fruits could be closely related to the increase in vigor conferred by the RS 842 commercial hybrid rootstock, which in turn increased plants’ water and nutrient absorption; this in turn improved the plant’s capacity to sustain the development and ripening of a higher number of fruits compared to plants grafted on less vigorous rootstocks. Similarly, Rouphael et al. [30] reported that a specific rootstock–scion combination could modulate hormonal balance, influence sex expression, and reduce fruit abortion. However, our research pointed out that the response is highly dependent on the rootstock–scion combination.

Grafting onto RS 842 commercial hybrid rootstock significantly enhanced the average weight of marketable fruits. There are reports confirming that fruit weight in cucurbits is positively affected by rootstocks [31,32]. Similarly, Zhang et al. [33], evaluating the effects of grafting on three different watermelon cultivars, report that fruits from grafted plants have higher weight than those harvested from non-grafted plants [33]. In our study, watermelon harvested from plants grown during the second year had the highest average fruit marketable weight compared to those obtained from plants cultivated during the first year. We hypothesize that this effect is linked to the lower sink–source competition, which is related to the more favorable environmental conditions observed during the second cultivation year [34].

### 3.2. Fruit Qualitative Features

Our outcomes on pulp and peel firmness are in contrast with those of Suárez-Hernández et al. [35], who evaluated the effect of watermelon grafted onto *Lagenaria siceraria* and reported an 85% increase in epicarp firmness when watermelon was grafted onto *L. siceraria*. The inconsistent results highlight a strong correlation between firmness and the employed rootstock. Grafting watermelon onto interspecific pumpkin hybrids allows the modulation of several genes that regulate the primary and secondary metabolism of the plants [5]. During the second cultivation year, the effect of LS may have indirectly influenced cell wall deposition, improving the uptake of calcium and other structural elements involved in the stability of plant tissues [36,37]. Considering cultivation in the open field and therefore the impossibility of controlling temperature, humidity, rainfall, and solar radiation, we hypothesize that these results are related to a different climatic trend between the two years examined. In our study, the highest values of peel firmness, mainly recorded in control plants grown during the second year, could be related to better growth conditions during the second year, which may have reduced the relative advantage of grafting and lignosulfonate. Watermelon fruits from grafted control plants grown during the first year had the highest pulp firmness. Although many studies indicate that the firmness of grafted watermelon pulp increases compared to fruits from non-grafted plants [38,39], there are also studies that report no significant variations [40,41]. In line with Bruton et al. [42] and Soteriou et al. [43], interspecific pumpkin hybrid rootstocks have a positive effect on pulp firmness. Devi et al. [38] also reported a significant increase in firmness when watermelon cv. Fujihikari is grafted onto *C. maxima* × *C. moschata* compared to fruits from plants grafted onto *L. siceraria*. Similar results were highlighted by Bruton et al. [42] on diploid and triploid watermelon grafted on hybrid rootstocks of *C. ficifolia* and *C. maxima* × *C. moschata*, highlighting the highest values in terms of pulp firmness. These studies show that (i) grafting has a direct effect on fruit texture, and (ii) the effects on texture are strongly influenced by the type of rootstock employed. Therefore, choosing the right graft/rootstock combination is a crucial element of improving fruit quality and post-harvest shelf-life.

Results on epicarp thickness are particularly relevant for post-harvest fruit shelf-life, as a higher peel thickness often guarantees a longer shelf-life of the product and greater resistance to transport operations along the entire supply chain [44]. A different pulp/skin ratio is hypothesized among grafted, self-grafted, and non-grafted plants treated or not with LS. Our results are partially in line with the findings of Alan et al. [45], who demonstrated that the epicarp of the cvs. ‘Crimson Tide’ and ‘Crimson Sweet’ grafted onto *Lagenaria siceraria* was significantly thicker than that of fruits from ungrafted plants. This could confirm a higher pulp/skin ratio in some scion–rootstock combinations compared to non-grafted plants.

The variation observed in watermelon fruit dry matter suggests a strong interaction between environmental conditions, grafting, and biostimulant application. On average, the highest values were recorded in fruits from plants grown during the second year. In particular, the highest values were reported in fruits from LS-treated grafted plants grown during the second cultivation cycle. Considering the open-field cultivation, environmental factors (particularly water availability) likely played a major role, while grafting may have affected the plants’ water uptake and biomass distribution.

Our results on fruit dry matter data are in agreement with those of Aslam et al. [46], who reported a dry weight decrease in cucumber grafted on *Cucurbita moschata* rootstock as compared to non-grafted plants. Our results are also in line with those of Ertani et al. [25], who reported an increase in dry matter in plants supplied with lignosulfonate compared to non-biostimulated plants. The same authors report an increase in GS and GOGAT enzymatic activity, which could be involved in biomass allocation and fruit composition, including dry matter content. Biostimulant application increased dry fruit matter. This result could be related to improvement in nitrogen, phosphorous, and potassium absorption and to a hormone-like effect capable of increasing root system development and ultimately nutrient uptake efficiency [47].

Soluble solid content is considered a key quality indicator of fruit’s sweetness [38]. Several authors have reported that the rootstock–scion combination could affect SSC content [39,48,49]. As reported by Devi et al. [38], SSC values differed when two different watermelon cultivars are grafted onto the same rootstock (*Cucurbita maxima* × *Cucurbita moschata*), indicating that the rootstock–scion combination had a stronger influence. In contrast, additional studies have not shown a significant effect of grafting on SSC when watermelon is grafted onto *L. siceraria* rootstocks [30,50]. Moreover, our results showed that the LS has an impact on SSC content. This outcome could be related to the effect of LS on plant metabolism. In this regard, Ertani et al. [25] showed an increase in RuBisCO activity, a key enzyme responsible for CO_2_ fixation in the Calvin cycle which is closely related to the photosynthetic efficiency of plants. Higher photosynthetic efficiency is linked to increased sugar biosynthesis, which leads to higher SSC values. The increase in RuBisCO activity in LS-treated plants could be due to the increase in the number of chloroplasts per cell, as proposed by Jannin et al. [51].

### 3.3. Carotenoid Content

In our study, grafting significantly increased carotenoid content. Yang et al. [52] showed that the content of some carotenoids increases significantly in fruits of grafted plants. Moreover, other authors have reported that grafting on interspecific hybrid squash–rootstocks significantly increases lycopene levels in watermelon [53,54]. Different graft combinations can guarantee more vigorous development and greater photosynthetic capacity, which affect the production of sugars and consequently their concentration into the plastids, where they participate in various metabolic pathways. High sugar levels promote upregulation of the methylerythritol phosphate (MEP) pathway. This pathway synthesizes intermediate precursors for the biosynthesis of several isoprenoids, including carotenoids [55]. Grafting generally increases the expression of the main genes involved in carotenoid biosynthesis, supporting a greater accumulation of pigments such as lycopene, β-carotene, and total carotenoids in fruits. These results suggest that grafting can positively modulate the carotenoid pathway, improving both the color and nutritional value of watermelon. Results also showed that the LS application significantly increased the content of β-carotene, lycopene, and total carotenoids. As hypothesized by Ertani et al. [25], the lignosulfonate mechanism could be like that of humic substances. The study outcomes are in line with Dawood et al. [56], who observed an increase in carotenoids in fava bean plants treated with humic acid compared to control plants. As reported by Bayat et al. [57], humic acid can increase chlorophyll and carotenoids biosynthesis due to an increase in nutrient uptake. This hypothesis is supported by Mezeyová et al. [58], who evidenced that humic acid-based biostimulants promote nutrient availability in plants.

### 3.4. Colorimetric Traits

In our research, we hypothesized that the positive effects of LS on watermelon fruit lightness could be linked to an increase in plant water uptake during the first year [25], which in turn influenced fruit hydration, allowing for a shinier surface compared with less hydrated fruits [59]. Regarding grafting effects, our outcomes are in contrast with those of Kyriacou et al. [18], who reported that the fruits of the grafted plants have a lower lightness value than those of ungrafted plants. Moreover, Suárez-Hernández et al. [35], comparing L* values in watermelon fruits harvested from grafted plants with those from non-grafted plants, demonstrated an increase in lightness. These contrasting values may be attributed to the different scion–rootstock combinations that could affect the cellular turgor and water content in fruits [38]. On average, grafting increased the redness of watermelon pulp compared to fruits harvested from non-grafted or self-grafted plants of both cultivation years. Similarly, Kyriacou et al. [60], studying the effect of grafting on quality features in watermelon fruits, found that grafting significantly increased redness values in watermelon fruit pulp. The highest a* values in grafted plants could be related to lycopene content in fruits. As reported previously, lycopene is a key pigment for watermelon, providing the pulp’s red color. Grafting directly affected lycopene content, determining higher a* values in fruits from grafted plants. This study revealed higher yellowness in fruits from grafted plants compared to those from non-grafted or self-grafted plants. These results could be related to the higher content of carotenoids recorded in grafted plants, which influenced the yellow color of the pulp.

### 3.5. Stomatal Conductance and Transpiration

The highest values in terms of stomatal conductance and transpiration were recorded in self-grafted control plants and in non-grafted and self-grafted control plants, respectively. The increase in water retention of grafted plants compared to non-grafted or self-grafted plants is often linked to an increase in the net rate of CO_2_ assimilation, a reduction in the transpiration rate, or both [61]. In a study on cucumber grafted on *L. siceraria*, the increase in water efficiency compared to the self-grafted cucumber was the result of a higher CO_2_ assimilation rate and a lower transpiration rate compared to the self-grafted plants [62]. Rootstock-induced changes in stomatal development affect water retention in grafted plants, as reduced transpiration is linked to lower stomatal density in grafted plants [62]. LS application reduced stomatal conductance and transpiration in plants, contributing to a better water balance. Considering the high water requirements of watermelon and the average temperatures recorded in the cultivation period, it is assumed that the grafted plants were subjected to a condition of mild water stress and that therefore the grafted and non-biostimulated plants manifested a lower stomatal conductance and transpiration rate in response to this stress in order to ensure greater efficiency in the use of water resources. Interestingly, the results also showed that when the biostimulant was administered, the highest results in terms of stomatal conductance and transpiration were recorded in grafted plants compared to non-grafted or self-grafted ones. These results could be linked to a pre-activation stress response due to the application of biostimulants. A recent study highlighted the effects of some elicitors and biostimulants that can optimize plant growth and enhance plant responses to abiotic stresses such as water stress [63]. The reduction in stomatal conductance in LS-treated plots and positive effect on yield features suggest an improvement in stomatal opening regulation. Thus, it seems that LS promotes more conservative use of water resources, which in turn affects plant physiological activity and production. However, since photosynthetic rate and water use efficiency were not recorded, this statement should be considered just one possible physiological interpretation.

## 4. Materials and Methods

### 4.1. Field Experiments and Vegetal Material

The experiment was carried out in the experimental field of the SAAF Department of the University of Palermo, Monreale (38°04′58″ N, 13°17′31″ E; 310 m a.s.l.), in the province of Palermo in Sicily, Italy, during the 2024 and 2025 growing seasons (northern hemisphere spring–summer) on Alfisol soil (USDA Soil Taxonomy, Soil Survey Staff. 2014) [64]. The experimental field was characterized by a medium-texture, alkaline pH (7.6), with 1.2 mg kg^−1^ of total nitrogen, 1.3 mg kg^−1^ of available phosphorous, and 241 mg kg^−1^ of exchangeable potassium. Daily temperatures and precipitation were obtained from a data logger (Trotec BL 30) located in the experimental plots. Watermelon (*Citrullus lanatus* (Thunb.) Matsum. & Nakai) seedlings were obtained from a commercial nursery. The commercial watermelon hybrid cv ‘Sentinel’ F_1_ was used as scion to obtain non-grafted plants (NG), self-grafted plants (SG) and plants grafted (G) onto an interspecific cucurbit rootstock F_1_ ‘RS 841’. Transplanting was performed on April 28 in both 2024 and 2025 growing seasons. All plots had an inter-row spacing of 3 m and a within row spacing of 2.5 m to reach a final density of 0.13 m^−2^. To complement natural rainfall and avoid water deficit, plots were drip-irrigated for the whole of the growing seasons for a total of 5,000 m^3^ ha^−1^. Water was supplied daily according to ETc, calculating ET_0_ through data from a weather station. The drip irrigation system was located under a mulch (transparent polyethylene film of 50 μm) utilized to avoid weeds. Plants were fertigated with 120 kg ha^−1^ of nitrogen, 120 kg ha^−1^ of phosphorous, 180 kg ha^−1^ of potassium, 80 kg ha^−1^ of calcium, and 30 kg ha^−1^ of magnesium, administered in three phases (20, 50 and 80 days after transplant). No pest management was required. The cultivation cycle for both years lasted 93 days.

### 4.2. Biostimulant Application and Rootstock Material

The biostimulant treatment (LS) was carried out using Statia^®^ (Mugavero Fertilizers, Termini Imerese, Palermo, Italy). This product is a lignosulfonate obtained through the sulfonation of lignin and contains 13.2% phosphorus pentoxide (P_2_O_5_), 8.4% potassium oxide (K_2_O), and 2.4% magnesium oxide (MgO). At 10 days after transplant and in 15-day intervals, plants were treated with 5 mL L^−1^ of Statia^®^ for the entire growing cycle (6 applications in total). The dose was applied following manufacturer’s recommendations. Biostimulant application was performed using 250 mL of biostimulant solution per plant applied via fertigation. Plants without the application of biostimulants were used as control.

The rootstock used was the interspecific hybrid F_1_ ‘RS 841’ obtained by the combination of *Cucurbita maxima* × *Cucurbita moschata*. It is widely used in horticultural systems due to its high graft compatibility, uniform fruit size, and precocity. In addition, it shows vigor and high productivity even under biotic stressors such as soil-borne pathogens (soil fungi and nematodes). Plants were grafted using the single-cotyledon technique.

### 4.3. Experimental Design and Statistical Analysis

The study consisted of six treatments in combinations of biostimulated or non-biostimulated and non-grafted (NG), grafted (G), or self-grafted (SG) plants in two different growing seasons (2024–2025). Each treatment was replicated three times; each replicate contained 10 plants, for a total of 180 plants per year of experimentation. Treatments were arranged in a randomized complete block design, with each block considered as replicate. A three-way analysis of variance (ANOVA) was performed, using grafting (G), biostimulants (LS), and year (Y) as the main factors and including their main effects and interactions on yield and quality parameters. When three-way interaction was not significant, subsequent two-way ANOVA enclosing all pairs of treatments (year × grafting, year × biostimulant and grafting × biostimulant) was performed. The components of variance were computed using the Type II sum-of-squares. The analysis was performed using IBM SPSS Statistic 28.0 (IMB Corp., Armonk, NY, USA). Mean values were compared using Tukey’s HSD test at a significance level of 0.05.

### 4.4. Physiological, Yield, and Quality Trait Measurements

Stomatal conductance and transpiration rate were assessed using a CIRAS-4 Portable Leaf Gas Exchange System (PP Systems, Amesbury, MA, USA) during the 2024 and 2025 growing seasons. The measurements were taken 60 days after transplanting, during the fruit ripening phase, on a clear day from 10 to 12 a.m. (Tamb: 35.6 ± 0.8 °C; Tcuv: 38.9 ± 0.9 °C; Tleaf: 37.2 ± 1.1 °C). Data were taken over the lamina of upper unfolded leaves of two random plants per replica, repeating the measurements three times per plant. Leaves were exposed to a photon flux density of 1200 µmol m^−2^ s^−1^. At physiological maturity, all plots were harvested, and total yield, marketable yield, non-marketable yield and average fruit weight of marketable fruits were recorded in all plants and expressed in kg plant^−1^ or kg fruit^−1^, respectively.

In parallel, five mature marketable fruits, randomly chosen per each biological replicate, were randomly harvested to measure quality traits in laboratory. Color, firmness, epicarp thickness, soluble solids content, and carotenoid content were determined using fresh fruit or fresh pulp. For dry matter determination, approximately 200 g of fresh pulp was collected from each sample, weighed, and subsequently lyophilized using a FreeZone Triad Freeze-Dryer (Labconco Corporation, Kansas City, MO, USA). The lyophilized samples were then stored for further analysis.

CIElab color parameters—lightness (L*), redness (a*) and yellowness (b*)—were measured in the central part of the fruit using a Chromameter CR-400 (Konica Minolta Sensing Europe, Milan, Italy).

Firmness was measured using a penetrometer (Trsnc, Forlì, Italy) and expressed in Newtons (N), and thickness of the epicarp was measured using a digital caliper (Logilink 150 mm—I-KL-150D) and expressed in millimeters (mm). To maintain the independence of assessments, the measurements were performed on opposite equatorial sides of the fruit for the peel and in the central and outer regions for the pulp.

Soluble solids content (SSC) was appraised on filtered fruit juice with the use of a digital refractometer (Hanna instrument, Padua, Italy).

Carotenoid content was spectrophotometrically determined according to the method proposed by Nagata & Yamashita [65]. In this method, 500 mg of fresh samples were vigorously shaken with 10 mL of acetone/hexane for 1 min and then filtered to obtain the pigments in the extract. The absorbance of the extracts was measured at 453, 505, 645, and 663 nm. β-carotene and lycopene content were estimated as follows (Formulas (1) and (2)):

β-carotene = 0.216 × A663 − 1.220 × A645 − 0.304 × A505 + 0.452 × A453(1)

lycopene = −0.0458 × A663 + 0.204 × A645 − 0.372 × A505 + 0.0806 × A453(2)

Results were expressed as mg of β-carotene and mg of β-lycopene in 100 gr of dry weight (DW). Total carotenoid was calculated as the sum of β-carotene and lycopene concentration. Thus, the reported data for total carotenoids represent the sum of these two compounds and not an independent measurement.

## 5. Conclusions

The use of sustainable strategies in open-field agriculture should not only increase or improve yields but also enhance organoleptic quality, with the aim of producing foods with high nutritional value that contribute to a balanced human diet. This work constitutes a first approach reporting how environmental conditions can modify the yield and quality of watermelon in the open field. Overall, data indicated that both LS and grafting could be considered effective strategies through which to improve watermelon performance. Both biostimulant and grafting improved yield and the qualitative and nutritional quality (in β-carotene, lycopene and carotenoids) of watermelon. Moreover, stomatal conductance and transpiration were reduced by the application of biostimulants, whereas grafting prompted a significant enhancement of stomatal conductance. Accordingly, this study suggested that for number of marketable fruits and average weight of marketable fruits, lycopene and carotenoids in the year of experimentation should not be taken into consideration, whereas for other parameters—such as percentage of fruit dry matter, solid soluble content, pulp firmness, and epicarp thickness—the year of experimentation should be taken into consideration in order to improve quality of this crop. The present study forms the basis of future studies involving different types of biostimulants and rootstocks, which will fill the knowledge gap regarding the combined use of these two agronomic tools in open-field conditions.

## Figures and Tables

**Table 1:** Effects of biostimulants, grafting, and year on the number of marketable watermelon fruits and the average weight of marketable fruits.

Treatments	No. of Marketable Fruits (No. plant^−1^)		Average Weight of Marketable Fruit (kg fruit^−1^)	
*Biostimulant* (*B*)				
Control	2.02	a	15.02	a
LS	2.04	a	15.47	a
*Grafting* (*G*)				
NG	1.93	b	14.68	ab
SG	2.03	ab	14.68	b
G	2.13	a	15.91	a
*Year* (*Y*)				
Y1	2.07	a	14.83	b
Y2	1.99	a	15.65	a

Different letters indicate different means according to Tukey’s HSD test at *p* ≤ 0.05. LS: lignosulphonate-based biostimulant; NG: non-grafted; SG: self-grafted; G: grafted; Y1; 2024; Y2: 2025.

**Table 2:** Effect of biostimulant, grafting, and year on watermelon pulp b* color parameters.

Treatments	b* Pulp	
*Biostimulant*		
Control	20.50	a
LS	20.50	a
*Grafting*		
NG	19.08	b
SG	18.92	b
G	23.50	a
*Year*		
Y1	22.83	a
Y2	18.17	b

Different letters indicate different means according to Tukey’s HSD test at *p* ≤ 0.05. LS: lignosulphonate-based biostimulant; NG: non-grafted; SG: self-grafted; G: grafted; Y1; 2024; Y2: 2025.

**Table 3:** Effect of biostimulant, grafting, and year on watermelon lycopene and carotenoid concentration.

Treatments	Lycopene (mg 100g^−1^ dw)		Carotenoids (mg 100g^−1^ dw)	
*Biostimulant*				
Control	0.15	b	0.27	b
LS	0.19	a	0.34	a
*Grafting*				
NG	0.11	b	0.22	b
SG	0.13	b	0.24	b
G	0.25	a	0.46	a
*Year*				
Y1	0.15	a	0.29	a
Y2	0.18	a	0.32	a

Different letters indicate different means according to Tukey’s HSD test at *p* ≤ 0.05. LS: lignosulphonate-based biostimulant; NG: non-grafted; SG: self-grafted; G: grafted; Y1; 2024; Y2: 2025.

## Data Availability

The data that support the findings of this study are available from the corresponding authors upon reasonable request due to privacy.

## Supplementary Materials

The following supporting information can be downloaded at: https://www.mdpi.com/article/10.3390/plants15182887/s1, Table S1: Significance of the three-way analysis of variance for all recorded parameters.

## Abbreviations

The following abbreviations are used in this manuscript:

LS	Lignosulphonate
NG	Non-grafted
G	Grafted
SG	Self-grafted
Y1	Year 1
Y2	Year 2
Y	Year
