# Supplementary material for: Effect of Lignosulphonate-Based Biostimulants and Grafting on Watermelon Plant Performance Under Open-Field Conditions for Two Consecutive Years
Source: Plants (Basel). 2026 Sep 21;15(18):2887. doi: 10.3390/plants15182887 (PMC13611350; doi:10.3390/plants15182887)
Supplement: Supplementary file 1 [file plants-15-02887-s001.zip › plants-4533390-supplementary.pdf]

Table S1. Significance of three-way Analysis of Variance for all recorded parameters.

Parameters	Biostimulant (B)	Grafting (G)	Year (Y)	B × G	B × Y	G × Y	B × G × Y
Marketable yield (Kg plant^{-1})	**	***	NS	NS	*	NS	NS
No. marketable fruit (no. Plant^{-1})	NS	**	NS	NS	NS	NS	NS
Average weight of marketable fruit (Kg fruit^{-1})	NS	**	NS	NS	NS	NS	NS
L* pulp	NS	NS	NS	NS	***	*	NS
a* pulp	NS	***	***	NS	NS	***	NS
b* pulp	NS	***	***	NS	NS	NS	NS
Pulp firmness (N)	NS	*	***	***	***	NS	***
Peel firmness (N)	NS	NS	***	*	***	**	*
Epicarp thickness (mm)	NS	NS	NS	NS	NS	***	***
SSC (°Brix)	*	***	*	**	***	**	***
Dry matter (%)	NS	NS	***	***	***	NS	*
Stomatal conductance (mmol H2O m^{-2} s^{-1})	***	***	NS	***	NS	*	NS
Transpiration (mmol H2O m^{-2} s^{-1})	***	NS	NS	***	NS	NS	NS
β-carotene (mg 100g^{-1} dw)	***	***	NS	***	NS	NS	NS
Lycopene (mg 100g^{-1} dw)	*	***	NS	NS	NS	NS	NS
Carotenoids (mg 100g^{-1} dw)	***	***	NS	NS	NS	NS	NS

NS: not statistically significant; *: significant at $p \leq 0.05$; **: significant at $p \leq 0.01$; significant at $p \leq 0.001$.
